# Novel Silicone-Grafted Alginate as a Drug Delivery Scaffold: Pharmaceutical Characterization of Gliclazide-Loaded Silicone-Based Composite Microcapsules

**DOI:** 10.3390/pharmaceutics15020530

**Published:** 2023-02-04

**Authors:** Ahmed Gedawy, Giuseppe Luna, Jorge Martinez, Daniel Brown, Hani Al-Salami, Crispin R. Dass

**Affiliations:** 1 Curtin Medical School, Curtin University, Bentley 6102, Australia; 2 Curtin Health Innovation Research Institute, Curtin University, Bentley 6102, Australia

**Keywords:** silicone, alginate, drug delivery, PDMS, microcapsule, ionic gelation

## Abstract

A novel gliclazide-loaded elastomeric carbohydrate pharmaceutical vehicle was successfully developed. This new siliconized alginate platform showed pseudoplastic rheology with a zeta potential ranging from (−43.8 mV to −75.5 mV). A Buchi-B390 encapsulator was employed to formulate different types of silicone-grafted alginate microcapsules loaded with gliclazide relying on the vibrational ionic gelation technology. The use of tetraethyl orthosilicate (TEOS) to crosslink the silicone elastomer (hydroxy terminated polydimethylsiloxane) of this new platform had improved the gliclazide encapsulation (>92.13% ± 0.76) of the free-flowing composite microcapsules, which showed good mechanical durability (up to 12 h in PBS pH 6.8) and promising results to sustain the drug release.

## 1. Introduction

The versatile film-forming ability of sodium alginate biopolymer is well-documented either alone or with other polymers and has been employed in many industries including the food industry, pharmaceutics and tissue engineering [1,2,3,4,5,6,7]. Crosslinking of sodium alginate with divalent cations is the predominant technique employed to obtain a mechanically stable film and to entrap different therapeutics within the ionotropically gelled 3D scaffold [2,3,4,8]. Silicone polymers known as polydimethylsiloxane (PDMS) are another inert, biocompatible class of polymers that has been safely employed in biomedical applications, microfluidics, contact lenses, catheters, cardiac pacemakers, coatings for cochlear implants and soft tissue substitutes [9,10,11,12,13]. Silicone elastomers have also been used in various drug delivery systems for hormones [14], antibiotics [15], anticancer therapy [16], anticholinergics [17], analgesics [18] and to sustain the delivery of ophthalmic preparations [19]. 

Modification of silicone elastomers is often required to sustain or control the drug release characteristics [10] and can be achieved by various techniques such as functionalization of PDMS, copolymerization or blending with another polymer, through interpenetrating polymeric network or through surface coating with PDMS [10,20]. The literature outlined different methods to crosslink silicone elastomers such as peroxide treatment, irradiation, condensation and addition to facilitate the use of silicone coats/films [10]. The use of platinum or tin in some of these methods could be a serious limitation in its use for drug delivery systems due to safety concerns [10,11]. Surfactant-mediated crosslinking of PDMS is a pharmaceutically acceptable alternative to obtain a silicone coat that has been employed in tablet coating to control the drug release [21,22,23,24,25]. In tablet coating with silicone elastomers, the hydrophilic polyethylene glycol (PEG) or polyvinyl pyrrolidine (PVP) were often used to facilitate the drug release from the tablet core through channelling through the PDMS film [21,22,23] [26,27]. An integrated or interlinked platform utilizing both PDMS and sodium alginate could be a pharmaceutically conceivable goal to attempt to encapsulate different therapeutic agents relying on the characteristics of both polymers [28]. However, the hybrid film/coat of silicone elastomers with alginate is yet to be developed, characterized and improved. 

Tetraethylorthosilicate (TEOS) is an alkoxysilane that has a silicone backbone similar to that of PDMS [29,30]. TEOS hydrolyses in the presence of mineral acids such as phosphoric acid and hydrochloric acid as well as organic acids (acetic acid), where -OH groups will substitute alkoxyl groups attached to the silicone (Si) atom [29,30]. Acid catalysed hydrolysis of TEOS is generally fast [29] and is followed by a condensation reaction to form a siloxane bridge (-Si-O-Si-) [29,31]. The interaction between hydroxyl-terminated PDMS and TEOS in coatings has been noted in many industries [31,32,33,34] and has been used in pharmaceutical coatings, with some papers suggesting the use of sodium dodecyl sulphate (SDS) to mediate and facilitate such a reaction [21,22,23,24,25]. 

Gliclazide is a sulfonyl urea (second generation) that has been used for the management of glucose levels in type 2 diabetes patients [35,36,37,38,39]. Gliclazide is a hydrophobic and acidic drug that exhibits pH-dependent solubility (higher solubility in alkaline pH) [36,40,41]. The Biopharmaceutical Classification System (BCS) recognizes gliclazide as a class II therapeutic agent characterized by high permeability and low solubility, and the significant inter-subject variation is attributed to gliclazide erratic absorption, where the gliclazide maximum plasma concentration is achieved within a rather wide range (2–8 h) following oral administration [35,36,37]. Beyond its glycaemic effects, gliclazide also has antioxidant, vascular benefits and neuroprotective effects in diabetic peripheral neuropathy [36,42,43,44,45]. Microparticles (microcapsules) are designed to control or target the drug release of their payload to a certain tissue or organ in a preprogramed fashion [46,47]. The previous literature has outlined various polymers employed in the encapsulation process of many therapeutics including gliclazide [36,37,38,39,46,47]. The current study aims to formulate a novel PDMS-grafted alginate platform to encapsulate gliclazide as a drug model by jet nozzle vibration (Buchi B390 encapsulator (Buchi Labortechnik, Flawil, Switzerland)) and to characterize the new platform as well as the produced microcapsules.

## 2. Materials

Hydroxyl-terminated polydimethylsiloxane, PDMS (CAS 70131678); tetraethyl orthosilicate, TEOS (CAS 78104); sodium dodecyl sulphate, SDS (CAS 151213); low viscosity sodium alginate (CAS 9005383) and gliclazide (98%, CAS 21187984) were purchased from Sigma-Aldrich (St. Louis, MO, USA). Anhydrous calcium chloride (96%) was obtained from ThermoFisher Scientific (Melbourne, Australia). All other reagents and chemicals were of HPLC grade.

## 3. Preparation and Formulation of Gliclazide-Loaded Polymeric Dispersion 

### 3.1. Preparation of Control_N1, N2 and N3

**Figure 1:**
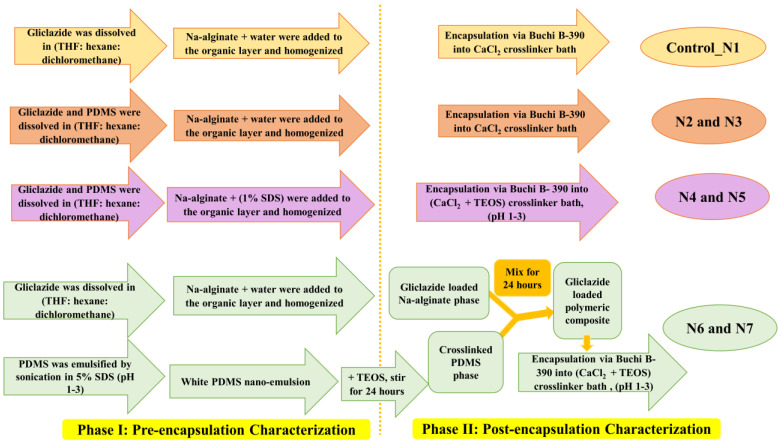
Formulation layout of different microcapsules.

Gliclazide (in case of control_N1) or gliclazide and PDMS (in case of N2 and N3) were dissolved in the least amount of an organic mixture (tetrahydrofuran, THF:hexane:dichloromethane at a 1:1:1 ratio) (Figure 1), then the specified amounts of sodium alginate and water (Table 1) were added to the organic layer and homogenized with (Ultra-Turrax homogenizer, Germany) for 5 1 min cycles each 30 s apart. The polymer to drug ratio was maintained at 2:1 in all formulations (Table 1). 

### 3.2. Preparation of N4 and N5

Gliclazide and PDMS were dissolved in the least amount of the same organic mixture (THF:hexane:dichloromethane at 1:1:1 ratio), the specified amount of alginate and 100 mL of 1% SDS was added and the samples were homogenized the same way for 5 cycles. Then, 50 mL of water was added and the samples homogenized for 1 more minute to maintain the same final dispersion volume (Table 1 and Figure 1).

### 3.3. Preparation of N6 and N7

PDMS was emulsified by sonication in 50 mL 5% acidified SDS (pH 1–3) for 15 min using a Bransonic ultrasonic cleaner (Brookfield, CT, USA) followed by 1 more minute using a UP200S probe sonicator (Teltow, Germany) to obtain a white silicone nanoemulsion to which an equal amount of TEOS was added to crosslink it by stirring for 24 h (crosslinked PDMS phase). Gliclazide was dissolved in the least amount of the organic mixture (THF:hexane:dichloromethane) and homogenized with alginate in 100 mL water (gliclazide-loaded alginate phase). Both phases were mixed and stirred for 24 more hours (Table 1 and Figure 1). 

### 3.4. Preparation of Gliclazide-Loaded Microcapsules

The previously prepared polymeric dispersions were extruded through a vibrational jet nozzle encapsulator (Buchi B-390, Switzerland) into 5% CaCl_2_ for control, N2 and N3 and into 5% CaCl_2_ and 5% TEOS, at pH range 1–3 for N4, N5, N6 and N7 to obtain spherical microcapsules. All microcapsules were left in their crosslinking bath for 15 min to ensure complete curing of the ionic gelation reaction, then decanted, washed with HPLC grade water, dried for 7 days in hot room (37 °C) and stored in a desiccator for subsequent experiments.

## 4. Phase I: Pre-Encapsulation Characterization of Gliclazide-Loaded Polymeric Dispersion 

### 4.1. Zeta Potential

A Zetasizer Nano ZSP (Malvern Instruments, Malvern, UK) was used to measure the electrochemical stability of all gliclazide-loaded polymeric dispersions. Measurements were performed in triplicate by dilution of few drops of each formulation with HPLC grade water at room temperature as per the laboratory protocol, recorded through Zetasizer software and presented as mean ± SD. 

### 4.2. Rheological Studies

The rheological features, shear rate, shear stress and average viscosity of gliclazide-loaded polymeric blends were determined via a Bohlin Visco 88 viscometer (Malvern Panalytical, Malvern, UK) in triplicates at different speeds at room temperature and presented as mean value ± SD.

### 4.3. Surface Active Properties

A Sigma 703 Tensiometer (Biolin Scientific, Tokyo, Japan) was used to determine the mean surface tension of all formulations (*n* = 3, ± SD) by recording the force needed to detach a thin film within a circular ring immersed in gliclazide-loaded formulations. 

### 4.4. Silicone Nanoemulsion Characterization (for N6 and N7)

The Dynamic Light Scattering technology (DLS) of the Zetasizer Nano ZSP (Malvern Instruments, Malvern, UK) was used to measure the particle size as well as the zeta potential of the SDS emulsified PDMS prior to its crosslinking with TEOS. Data are presented as mean of *n* = 3, ± SD.

### 4.5. Characterization of N6 and N7 Films after Crosslinking with TEOS

The crosslinked PDMS latex was dried in an oven at 80 °C for around 3–5 h till a film formed. Films from crosslinked N6 and N7 latices were characterized versus pure PDMS by scanning the spectrometric chart between 4000 cm^−1^ and 450 cm^−1^ using Fourier transform infrared spectroscopy (FTIR, Perkin Elmer, Waltham, MA, USA) and comparing the thermograms produced by ramp heating from 20 °C to 300 °C at 20 °C/min using differential scanning calorimetry (NETZSCH DSC 3500 Sirius, Munster, Germany) under 20 mL/min nitrogen purge and compared with an empty aluminium pan (control). The solubilities of the films were also tested in PBS pH 6.8 for 7 days. 

## 5. Phase II: Post-Encapsulation Characterization of Gliclazide-Loaded Microcapsules

### 5.1. Optical Microscopy and Particle Size Distribution

Against a dark background, the morphology and diameter of a few representative wet microcapsules of all formulations were determined with a Toupcam 14 MPA camera and Toupview software attached to a Nikon SMZ800 stereo optical microscope (Nikon, Melville, NY, USA). The uniformity of the formulated microcapsules and their particle size distribution curves were detected by the hydrodynamic diffraction of a laser beam emitted from a Mastersizer 2000 (Malvern Instruments, UK). 

### 5.2. Drug Loading and Encapsulation Efficiency

In triplicate determinations, an accurately weighed 100 mg of all formulations was placed into a 100 mL volumetric flask and the final volume was made up to 100 mL using PBS pH 7.4. These flasks were sonicated for half an hour then stirred at 37 °C for 24 h by the aid of a multishaker PSU 20 operated at 150 rpm. The samples were centrifuged and syringe filtered for HPLC determination of gliclazide content at 227 nm using a validated and reported HPLC method [40,41]. Gliclazide loading and encapsulation efficiency (EE%) were calculated by Equations (1) and (2), respectively, and are presented as mean ± SD [36].

(1) $$Gliclazide\;loading\;\%=\frac{Weight\;of\;gliclazide\;in\;microcapsule\;sample}{Weight\;of\;microcapsule\;sample\;}\times 100$$

(2) $$EE\;\%=\frac{Practical\;gliclazide\;weight}{Theoretical\;gliclazide\;weight\;}\times 100$$

### 5.3. Microcapsule Durability/Mechanical Resistance

The mechanical strength of ten microcapsules (in triplicate determination) from all formulations were placed in a 50 mL flask of deionized water, normal saline solution and PBS solution pH 6.8. Test flasks were exposed to a mechanical stress through a multishaker PSU 20 operated at 37 °C and 150 rpm [28]. The microcapsule durability % was determined by visual counting the average intact microcapsules (*n* = 3) that remained after shaking compared with the initial count and calculated by Equation (3) and presented as mean ± SD.

(3) $$Microcapsule\;durability\;\%=\frac{intact\;microcapule\;count\;after\;shaking}{initial\;microcapsule\;count\;}\times 100$$

### 5.4. Microcapsule Swelling

A dissolution apparatus (Erweka DT6, Heusenstamm, Germany) operated at 37 °C and 50 rpm was used to study the swelling behaviour of 100 mg of every formulation placed in a dissolution basket immersed in 0.1 N HCl pH 1.2 and PBS pH 7.2. At predetermined time intervals, swollen microcapsules were collected and surface media droplets were removed using a paper towel. The correlation between swollen and initial dry microcapsules was established through calculation of the average swelling index of every formula (*n* = 3) Equation (4) and presented as mean ± SD [37].

(4) $$Swelling\;index\;\%=\frac{swollen\;microcapsule\;weight-initial\;microcapsule\;weight}{initial\;microcapsule\;dry\;weigh}\times 100\;$$

### 5.5. Compressibility Index and Hausner Ratio

The compressibility and flow properties of formulated microcapsules were determined by measuring the volume occupied by two grams of every formula (in a glass measuring cylinder) before and after tapping on a bench 100 times to obtain the bulk and tapped densities, respectively. The average (*n* = 3) compressibility index (Carr’s index) and Hausner’s ratio can be calculated from Equations (5) and (6), respectively, and are presented as mean ± SD [48,49].

(5) $$Carr’s\;index\;\%=\frac{tapped\;density-bulk\;density}{tapped\;density}\times 100\;$$

(6) $$Hausner\;ratio=\frac{tapped\;density}{bulk\;density}\;$$

### 5.6. Gliclazide In Vitro Release 

A sample microcapsule of every formula equivalent to 100 mg of gliclazide was placed in 900 mL of PBS pH 7.4 (*n* = 3) of a dissolution apparatus (Erweka DT6, Germany) operated at 37 °C and with a 100 rpm paddle. At predetermined time intervals, 5 mL was sampled (syringe filtered for HPLC determination of released gliclazide by a validated HPLC method at 227 nm [40,41]) and 5 ml fresh PBS pH 7.4 was added to maintain sink conditions. Gliclazide powder (100 mg) was tested in the same way as a reference for the in vitro dissolution; data are presented as mean ± SD [38]. 

### 5.7. Fourier Transform Infrared Spectroscopy (FTIR)

A FTIR spectra (range 4000 cm^−1^ to 450 cm^−1^) spectrophotometer (Perkin Elmer, USA) was used to scan sodium alginate, gliclazide, blank microcapsules, control microcapsules, N2, N4 and N6 to measure the chemical stability of the gliclazide contained in the formulated microcapsules. 

### 5.8. Differential Scanning Calorimetry (DSC) 

Under 20 mL/min nitrogen purge and compared with the control empty aluminium pan, the thermograms of PDMS, gliclazide, sodium alginate, physical mixtures, blank microcapsule and formulated microcapsules were recorded by ramp heating of 5 mg of sample contained in a sealed aluminium pan (from 20 °C temp to 300 °C at 20 °C/min rate) using differential scanning calorimetry (NETZSCH DSC 3500 Sirius, Germany). 

### 5.9. Scanning Electron Microscopy (SEM) and Energy Dispersive X-ray (EDX)

With a suitable magnification, an SEM (Tescan MIRA3 XMU, Brno, Czech Republic) with an electron beam of 5-kV was used to scan and record the electron micrographs of a few platinum-coated microcapsules of every formula mounted on a round small glass stub. Aztec software (Oxford Instruments, Abingdon, UK) connected to an EDX Oxford X-Max^N^ 150 SDD X-ray detector (Oxford Instruments) was used to capture the topographic and qualitative elemental analysis of the surface of different microcapsules. 

## 6. Statistical Analysis

GraphPad Prism software (GraphPad Inc., version 5) was used for graphical presentation and the statistical analysis of mean ± SD triplicate data using raw means/totals and one way ANOVA considering *p* < 0.05 as statistically significant.

## 7. Results

### 7.1. Zeta Potential

**Figure 2:**
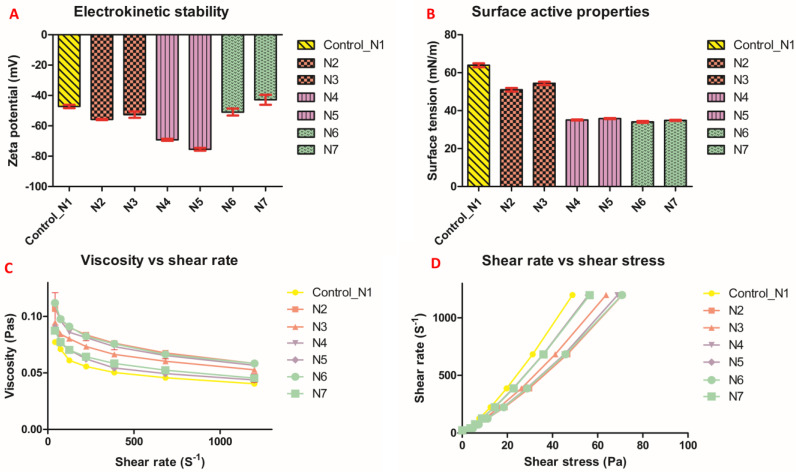
Zeta potential (**A**), surface tension (**B**) and rheological properties (**C**,**D**).

All formulated polymeric vehicles exhibited high electrochemical stability ranging from −43.8 mV to −75.5 mV compared with the control of −47.2 mV ± 1.12 (Figure 2A).

### 7.2. Rheological Features

The viscosity of polymeric vehicles of all formulations decreased with increased shear rate at different speeds in a non-linear fashion (Figure 2C). The relationship between shear stress and shear rate presented in Figure 2D confirmed the shear thinning and non-Newtonian behaviour of this PDMS-grafted alginate blend.

### 7.3. Surface Tension

The control polymeric vehicle had a surface tension of 63.9 mN/m ± 1.01. All other formulations had much lower surface tension. N3 had a surface tension of 54.4 mN/m ± 0.8 and N6 had the lowest surface tension of all, 34.1 mN/m ± 0.46 (Figure 2B).

### 7.4. Silicone Nanoemulsions Employed in N6 and N7 Formulations

**Figure 3:**
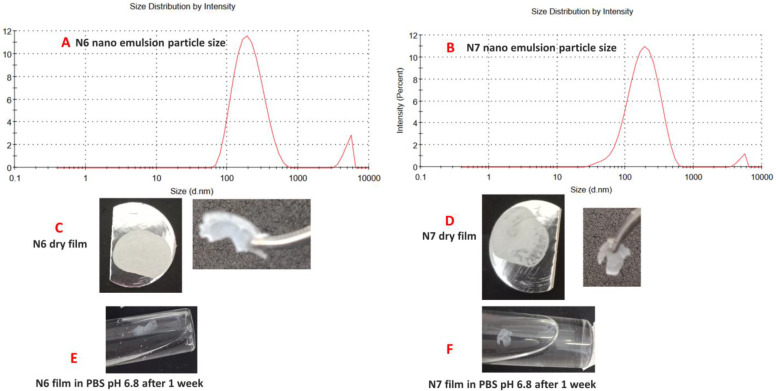
Size characterization for N6 nanoemulsion (**A**) and N7 nano emulsion (**B**). Images of N6 dry film (**C**). Images of N7 dry film (**D**). N6 film in PBS (**E**). N7 film in PBS (**F**).

The white PDMS nanoemulsions employed in N6 and N7 microcapsules showed zeta potentials of −82.3 ± 3.7 mV and −92.1 ± 3.5 mV, respectively, with a particle size of 201.5 nm size ± 3.3, polydispersity index of 0.35 for N6 latex (Figure 3A) and 171.6 nm size ± 2.8, polydispersity index of 0.26 for N7 latex (Figure 3B). 

### 7.5. N6 and N7 Crosslinked Films

**Figure 4:**
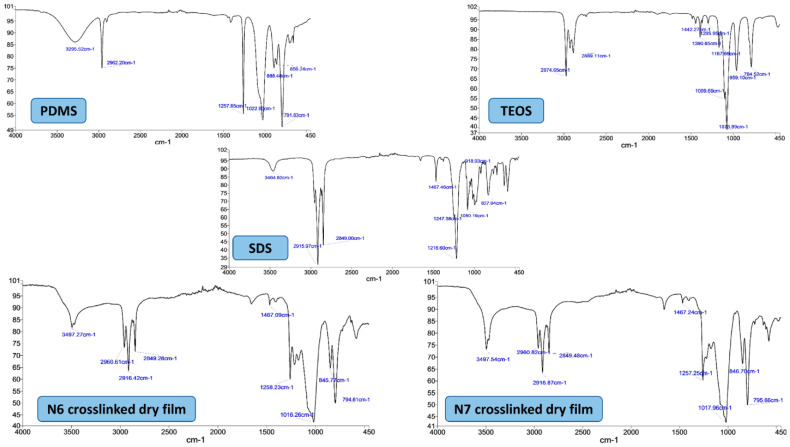
FTIR charts of PDMS (oil), TEOS (liquid), SDS (powder) and the crosslinked dry films of N6 and N7.

Figure 3C,D show the films produced after drying the TEOS crosslinked PDMS latexes. Table 2 summarizes the FTIR peaks identified for pure PDMS and TEOS. The disappearance of TEOS peaks in N6 and N7 films at 959.1 cm^−1^, 2974.6 cm^−1^ and 1099.6 cm^−1^ indicate the complete hydrolysis of TEOS and the conversion of its ester groups (-Si-O-Et) into active silanol (-Si-OH) [50]. The characteristic Si-OH groups of PDMS terminals at 3295.5 cm^−1^ and 888.4 cm^−1^ also disappeared in N6 and N7 films, suggesting their involvement in siloxane linkage -Si-O-Si- and their absence as free silanol groups due to an interaction between hydrolysed TEOS and PDMS [31,32,33]. Furthermore, the appearance of new peaks at 845.7 cm^−1^ and 846.7 cm^−1^ in N6 and N7 films and the increased intensity and the slight shift of the siloxane band at 1022.9 cm^−1^ of pure PDMS to a slightly lower wave numbers (1016.26 cm^−1^ or 1017.96 cm^−1^) in N6 and N7 films suggest a new type of siloxane bridge formation (-Si-O-Si-) between PDMS and TEOS due to crosslinking reaction [31,32,33]. The other peaks appearing in the N6 and N7 FTIR chart represent SDS peaks and the remaining PDMS functional peaks (Figure 4). 

**Figure 5:**
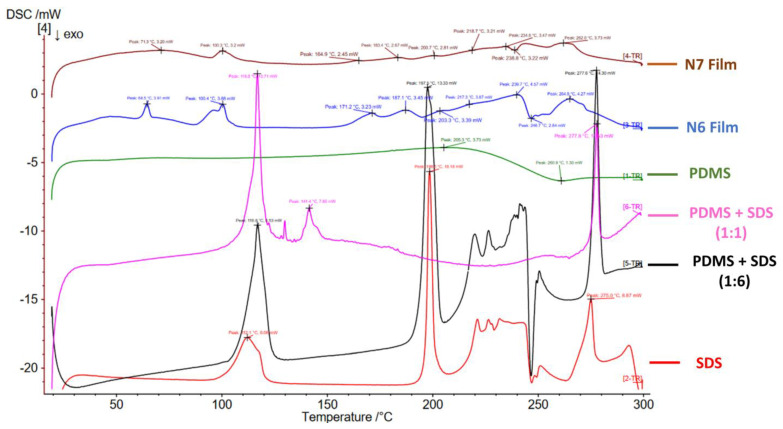
DSC thermograms of PDMS, SDS, physical mixtures of PDMS/SDS, N6 and N7 dry films.

The characteristic peaks of DSC thermograms of PDMS, SDS, N6 and N7 films are presented in (Table 3 and Figure 5). It seems that PDMS had a physical impact on the thermal behaviour of SDS, whereas the physical mixture of PDMS and SDS (1:1 ratio) did not have any of the peaks identified for PDMS. However, three endothermic peaks at 116.8 °C, 141.4 °C and 277.8 °C were observed (probably related to SDS). The physical mixture of PDMS and SDS (1:6) still did not show any peaks of PDMS; however, the peaks identified for pure SDS remained almost the same, especially the melting endothermic peak at 197.5 °C (Figure 5). The new peaks identified for N6 and N7 could be attributed to the crosslinked nature of the dry silicone films (Table 3 and Figure 5). Note that DSC testing of TEOS cannot be performed due to its flammable nature. N6 and N7 films showed some swelling but remained intact in PBS pH 6.8 after 1 week with occasional agitation (Figure 3E,F).

### 7.6. Optical Microscopy and Particle Size Distribution

**Figure 6:**
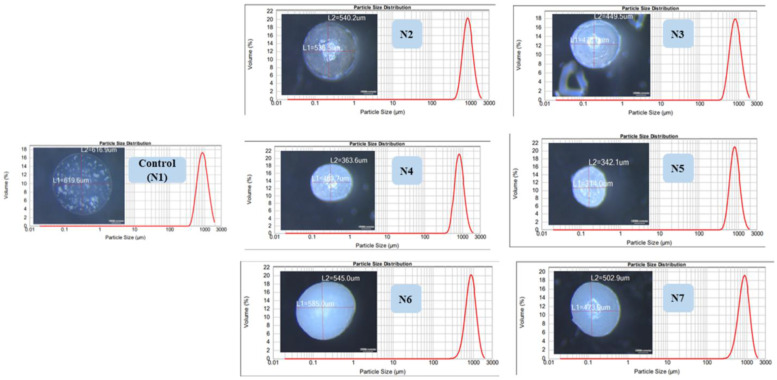
Microcapsule size by optical microscope and mastersizer.

Microscopic imaging revealed the spherical shape and the smooth surface of all wet microcapsules. The mastersizer results verified these findings and confirmed the uniformity of theses microcapsules (bell shaped, unimodal narrow distribution curves, span <1 with less than 0.3 deviation from the median size) (Figure 6). 

### 7.7. Gliclazide Loading and Entrapment Efficiency

**Figure 7:**
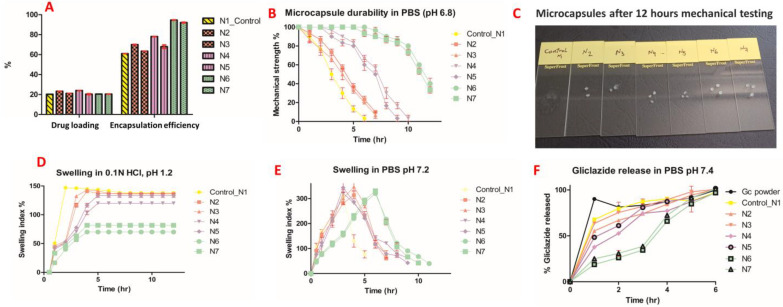
(**A**) Drug loading and encapsulation efficiency, (**B**) mechanical testing, (**C**) microcapsule images after 12 h of mechanical testing, (**D**) swelling in HCl, (**E**) swelling in PBS, (**F**) dissolution results.

Gliclazide loading of the presented formulations ranged from 20 to 24% compared with 20.3% ± 0.5 for the control. However, the encapsulation efficiency of all silicone formulated microcapsules exhibited a much higher percentage than the control (Figure 7A). 

### 7.8. Microcapsule Mechanical Durability

A slight swelling was noticed in formulated microcapsules as well as the control upon testing in deionized water for 24 h with no change in their counts. A total of 30% of the control microcapsules were ruptured in saline solution, whereas a slight swelling and no rupture was observed in the silicone-based microcapsules after 24 h. Only N6 and N7 formulations could withstand the mechanical stress test in PBS pH 6.8 for up to 12 h (Figure 7B,C). 

### 7.9. Microcapsule Swelling Behaviour

Much higher swelling indices for all silicone formulated microcapsules as well as the control were observed in PBS pH 7.2 than in acidic media over 12 h (Figure 7D,E).

### 7.10. Microcapsule Flow Properties 

All formulated microcapsules as well as control exhibited good flow properties where Carr’s index was in the range 9.23–12.12 and Hausner’s ratio was in the range 1.1–1.14 as per USP 37 (Table 1). N6 and N7 showed slightly higher densities than other formulations (based on volume occupied by same microcapsule weight).

### 7.11. Gliclazide In Vitro Release

All formulations showed a smaller percentage of gliclazide released in the first two hours compared with control microcapsules as well as gliclazide free powder. N6 and N7 exhibited the potential to control the release of gliclazide over 6 h better than the other microcapsules (Figure 7F).

### 7.12. FTIR of Microcapsules

**Figure 8:**
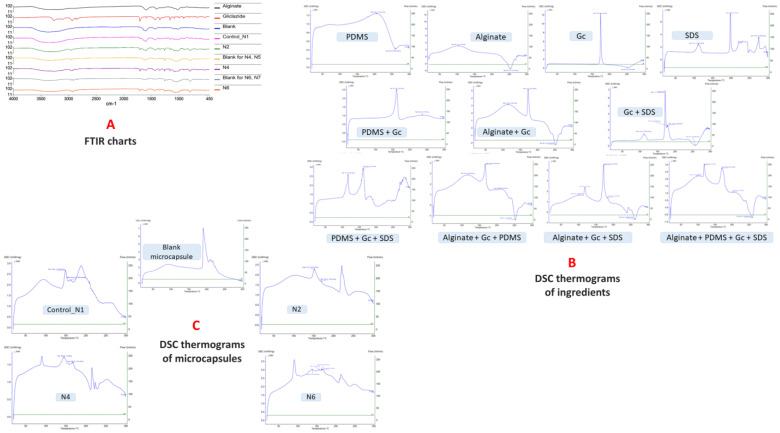
(**A**) FTIR of alginate, gliclazide and formulated microcapsules, (**B**) DSC thermograms of ingredients involved in the microcapsules and (**C**) DSC thermograms of microcapsules.

The characteristic FTIR peaks of gliclazide and Na–alginate are summarized in Table 2. No chemical interaction could be identified between gliclazide and any of the excipients used in the formulation where gliclazide exhibited all characteristic peaks essential for its pharmacological actions in all formulated microcapsules (Figure 8A). Due to the ionic gelation reaction and the formation of Ca–alginate salt, carboxylate peaks at 1594.75 cm^−1^ and 1406.41 cm^−1^ are shifted to 1602 cm^−1^ and 1427.2 cm^−1^, respectively, in all microcapsules [28,58]. The -OH peak of PDMS at 888.4 cm^−1^ disappeared in formulations crosslinked with TEOS and CaCl_2_ due to the interaction between TEOS and PDMS (we only tested high PDMS content formulations N4 and N6). However, the other -OH peak of PDMS at 3295.5 cm^−1^ cannot be monitored in the formulated microcapsules due to the broad alginate hydroxyl group appearing in the range 3359.06 cm^−1^–3241.7 cm^−1^ (Figure 8A). 

### 7.13. DSC of Microcapsules

DSC thermogram peaks for gliclazide, Na–alginate, N1, N2, N4 and N6 microcapsules are presented in Table 4. The endothermic peak of gliclazide is slightly shifted to a lower melting point range (161.6–172.7 °C) along with a decrease in its intensity when physically mixed with other excipients (Figure 8B). This could be attributed to the dilution effect or the physical interaction with the polymers used [36,37] without affecting the drug chemistry. The characteristic exothermic alginate peak at 253.1 °C disappeared in formulated microcapsules as well as control (Figure 8C). The changes to the endothermic position of the gliclazide peak could be attributed to the physical conversion of the drug into the non-crystalline state or even its dispersion into the polymeric platform and entrapment within the formulated microcapsules at the molecular level [56,57,59,60], where gliclazide can exist in three different conformers (closed, half extended or fully extended) [61].

## 8. SEM/EDX

**Figure 9:**
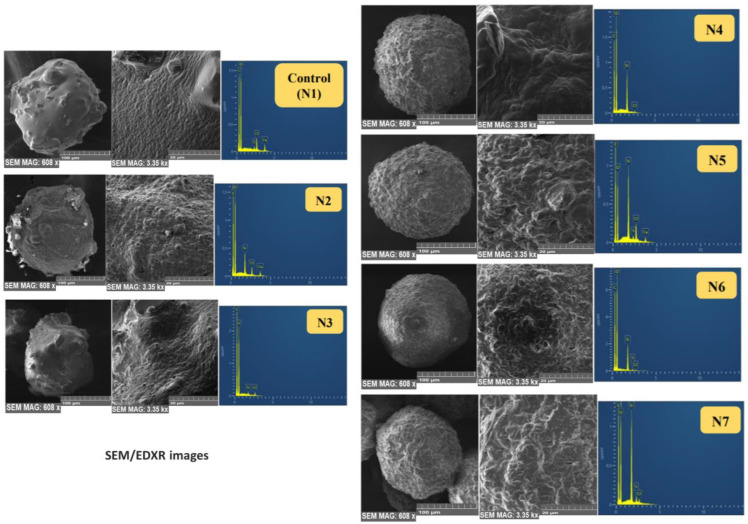
Scanning electron microscopic images of microcapsules (608× magnification, 100 µm scale for whole microcapsules and 3.35 kx magnification, 20 µm scale for microcapsule surfaces).

SEM micrographs revealed that the dry microcapsules are discrete nonporous spheres with an opaque continuous surface. EDX results showed an even distribution of the polymeric coat ingredient (C) carbon for alginate and (Si) silicone for PDMS (Figure 9).

## 9. Discussion

**Figure 10:**
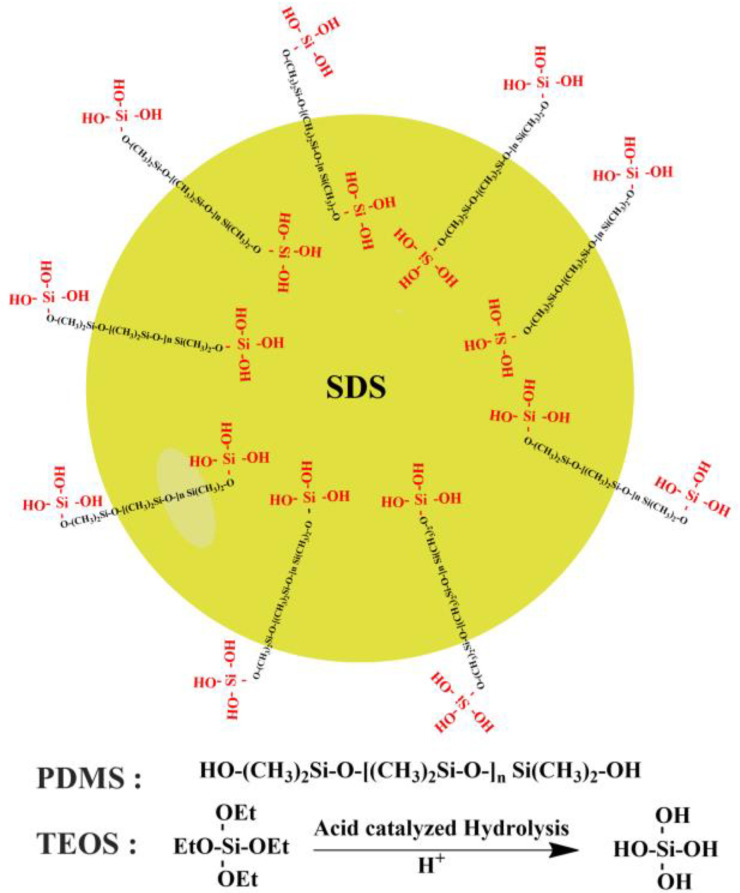
Schematic presentation of the crosslinking reaction between PDMS and TEOS assisted by SDS.

In this study, we have formulated different types of gliclazide-loaded microcapsules employing ionic gelation technology. We have explored and investigated the silicone grafting into a well-known biopolymer (sodium alginate) utilizing SDS in some formulations to study its effect on the polymeric blend pre-encapsulation as well as the behaviour of the new PDMS-grafted alginate platform post encapsulation. Crosslinking of silicone elastomers with alkoxysilanes was previously reported to be facilitated and mediated by surfactants such as sodium dodecyl sulphate (SDS) [21,22,23,24,25]. We attempted to double crosslink the heterogenous gliclazide-loaded polymeric platform (in the case of the N4 and N5 microcapsules) by two main reactions, the first is the interaction of alginate with calcium ions in the crosslinking bath (ionotropic gelation) and the second is the interaction of the PDMS of this platform with the alkoxysilane (TEOS) in the crosslinking bath. The later seems not to happen as quickly as the ionic gelation. For N6 and N7 formulations, silicone nanoemulsions were prepared by sonication with HCl acidified 5% SDS (Figure 1) and characterized (Figure 3A,B). Then TEOS was added (at a 1:1 ratio) and the sample was stirred on a magnetic stirrer for 24 h to crosslink the silicone elastomer prior to combining with the gliclazide-alginate phase. The hydrolysis of TEOS in the acidic pH of SDS emulsified PDMS latex was reported to facilitate its copolymerization with (-OH) terminals of PDMS due to its similarity with that of the silicone backbone through the formation of a siloxane bridge (Si-O-Si-) [29,31]. Figure 10 is hypothesized for the crosslinking reaction between PDMS and TEOS mediated by SDS and could explain the conversion of PDMS from the oily liquid state into the solid-state dry films (Figure 3C,D). N2 and N3 formulations were crosslinked with only CaCl_2_ to investigate and compare the silicone formulated microcapsules in absence of TEOS and SDS. 

Gliclazide-loaded polymeric dispersion exhibited high electrochemical stability in terms of the charge measured at the interfacial double layer of formulated heterogenous gliclazide colloidal system. Such a feature is required to ensure uniform distribution of gliclazide within the developed therapeutic vehicle. N4 and N5 showed the highest stability (−69.2 mV ± 0.6 and −75.5 mV ± 0.9, respectively), which could be attributed to the effect of SDS (extra repulsive and negative charge added) to homogenize gliclazide and the PDMS phase with alginate. This SDS effect on the zeta potential of N6 (−50.9 mV ± 2.3) and N7 (−42.8 mV ±3.3) was less pronounced, probably due to its consumption in the emulsification and crosslinking of PDMS with TEOS prior to consolidation with the gliclazide-loaded alginate phase (Figure 2A). SDS on the other hand augmented PDMS in reducing the surface-active properties (surface tension) of N4, N5, N6 and N7 dispersions compared with N1, N2 and N3 (Figure 2B), where low surface tension is a characteristic feature of PDMS [11]. Understanding the rheological properties of therapeutic vehicles is a significant pharmaceutical feature in predicting drug release behaviour [62,63]. Although all gliclazide-loaded polymeric blends exhibited non-Newtonian rheological characteristics, N2, N4 and N6 formulations with higher PDMS contents showed much higher viscosities (flow resistance) than N3, N5, N7 (with less PDMS) and control vehicles at all tested points at 25 °C. Decreased viscosities with increasing the force applied (shear rate) was a general trend of all tested polymeric fluids as well as the control sample. This pseudoplastic thixotropic rheological properties was also identified in the nonlinear (shear-thinning) relationship between the shear rate and shear stress (Figure 2C,D).

We noticed that microcapsules with higher PDMS contents (N2, N4 and N6) had a bigger microcapsule size than formulations with less PDMS (N3, N5 and N7), probably due to the higher viscosity of the N2, N4 and N6 formulations compared with the N3, N5 and N7 formulations. Although the control-loaded polymeric vehicle had the lowest viscosity of all dispersions, control microcapsules (N1) had the largest microcapsule size amongst all formulations. N2, N3, N4 and N5 microcapsules exhibited much smaller sizes than the control, probably due to hydrogen bonding between the -OH terminals of PDMS and the -OH or -COOH terminals of the Na–alginate structure [8]. N6 and N7 microcapsules did not exhibit such effects, presumably due to crosslinking of PDMS with TEOS prior to the addition and consolidation with Na–alginate (less chance of hydrogen bonding), in addition to the slightly higher viscosities they exhibit compared with other polymeric vehicles. N4 and N5 showed much smaller particle sizes than N2 and N3, probably due to the formation of a much more coherent PDMS-grafted alginate structure due to the emulsifying effect of SDS in N4 and N5, interaction of some PDMS molecules contained within the N4 and N5 polymeric vehicles with TEOS in the crosslinking bath, or shrinkage of Na–alginate blocks of the polymeric backbone into alginic acid due to the acidic pH of the crosslinker bath (Figure 6).

N2, N4 and N6 microcapsules with higher PDMS contents had higher gliclazide encapsulation efficiencies of 70.00 ± 0.5%, 78.01 ± 0.3% and 94.7 ± 0.4%, respectively, compared with 63.50 ± 0.2%, 67.94 ± 3% and 92.10 ± 0.8% for N3, N5 and N7, respectively (Figure 7A). These observations could be attributed to the integrated effect of the silicone-grafted alginate platform to enhance and improve the gliclazide entrapment in the formulated microcapsules compared with the silicone-free control microcapsules. The order of gliclazide entrapment could be presented as follows: N6 and N7 > N4 and N5 > N2 and N3 microcapsules. Such observations could be attributed to the stronger coating in N6 and N7 formulated with TEOS crosslinked PDMS. Another effect to consider is the acidic pH of the crosslinker bath used in N4, N5, N6 and N7, where the fraction of gliclazide (acidic drug, pKa 5.8) [40,64] anticipated to dissolve in the acidic crosslinker bath during the encapsulation process is reduced due to the lower solubility of gliclazide in acidic media [64]. 

Due to the hydrophobic nature of PDMS, formulated microcapsules exhibited minimal or no swelling in saline solution (0.9% NaCl) and deionized water compared with N1 (control), which enabled the silicone-formulated microcapsules to withstand mechanical testing in these media. Higher microcapsule durability and resistance to aqueous uptake was more pronounced in microcapsules with higher PDMS contents (N2, N4 and N6) than their counterparts with less PDMS (N3, N5 and N7). Generally, the swelling of all microcapsules in HCl was much less than in PBS. Such behaviour is governed by two factors, alginate shrinkage due to its conversion into alginic acid in acidic pH as well as the hydrophobic PDMS content in the microcapsules that makes the swelling order of the microcapsules as follows N1 > N3 > N2 > N5 > N4 > N7 > N6 (Figure 7D). In PBS pH 6.8 and PBS pH 7.2, microcapsules (Ca–alginate-grafted PDMS) behave differently where aqueous uptake is enhanced by loosening the alginate scaffold through an ionic exchange of its calcium ions with the sodium ions of PBS considering the hydrophobic nature of PDMS. Control microcapsules (N1) reached maximum swelling in PBS pH7.2 within 3 h, after which microcapsules started to break (Figure 7E); within 6 h of the mechanical testing in PBS pH 6.8, almost all N1 microcapsules disintegrated (Figure 7B). N2, N3, N4 and N5 microcapsules reached maximum swelling in PBS pH7.2 within 5 h (Figure 7E) and survived the durability testing in PBS pH6.8 for 7 h in the case of N2 and N3 microcapsules, whereas N4 and N5 were mechanically stronger and lasted for 10 h and 9 h, respectively, in pH 6.8 (Figure 7B). N6 and N7 microcapsules showed a delayed swelling peak maxima at 6 h in PBS pH 7.2 (Figure 7E) and had the strongest mechanical behaviour for up to 12 h in PBS pH 6.8 (Figure 7B,C). 

In PBS pH 7.4, 90.06% ± 0.61 of unformulated gliclazide (pure powder) was dissolved within the first hour (Figure 7F). Formulations with a high polymer content (N2, N4 and N6) released less gliclazide over 6 h compared with the control (N1) and formulations with low PDMS content (N3, N5 and N7), probably due to a stronger coating with higher PDMS content (Figure 7F). N4 and N5 formulations released less gliclazide than N2 and N3 over the dissolution time intervals. This could be attributed to the possible interaction between the PDMS of N4 and N5 drug-loaded polymeric blends and TEOS in the crosslinking bath facilitated by SDS [21,22,23,24,25] during the manufacturing process (Figure 1 and Table 1) or an extra coat of TEOS from the crosslinking bath that could have surrounded the formulated microcapsules through an interaction between the alkoxysilane, TEOS and alginate [65,66,67]. N6 and N7 microcapsules formulated with crosslinked PDMS showed a better tendency to sustain/prolong the gliclazide release over the dissolution study time compared with all other formulations, where less than 50% of the gliclazide was released in the first 3 h of dissolution. The inclusion of TEOS in the crosslinking bath of N4, N5, N6 and N7 seems to affect the mechanical durability of these microcapsules (Figure 7B,C) as well as the drug release profile (Figure 7F) compared with the use of CaCl_2_ alone in control (N1), N2 and N3 formulations. This could be attributed to the more hydrophobic surface of these microcapsules imparted by the water-soluble silicate produced from the hydrolysis of TEOS [66,67] and its permeation within the Ca–alginate PDMS network to form a colloid within this platform [65,66,67]. This effect could also be augmented by the presence of the crosslinked silicone polymer in N6 and N7 and could explain the dense rubbery appearance N6 and N7 microcapsules at the end of mechanical testing (Figure 7C) [66,67]. 

Control (N1) microcapsules showed an uneven and rough surface with cracks or small channels, probably due to alginate drying in addition to SEM drying and vacuuming. Surface composition analysis of N1 by EDXR revealed different levels of sulphur (S) on some sites (probably due to gliclazide migration to the surface) (Figure 9). All silicone-grafted microcapsules had a much more spherical shape than the control (N1) even after drying and vacuuming for SEM imaging. This property could be attributed to the elastomeric nature of PDMS to preserve the sphericity of the crosslinked microcapsules. Due to vacuuming and processing for SEM imaging, partial dissociation of PDMS from the polymeric network of N2 and N3 microcapsules was noticed and identified as surface bulges; N3 microcapsules (with less PDMS content) exhibited a less spherical shape and fewer surface bulges than the N2 formulated microcapsules in the same way but with much higher PDMS (Figure 9). This bulging effect and the partial dissociation of PDMS were absent in the surfaces of N4, N5, N6 and N7, which could be attributed to the homogenizing effect of SDS in the integration and consolidation of the silicone polymer within the sodium alginate network reflected on the microcapsule shape (more spherical) and the surface characterization of these microcapsules (even coating, bulge-free). EDXR analysis revealed that the surfaces of N2 and N3 did not exhibit any sulphur (S), reflecting that gliclazide migration to the surface could not be identified. Likewise, the N4 microcapsule surface did not reveal any sulphur content (S); however, some surface sulphur (S) was noticed in N5, N6 and N7, which presumably was due to SDS rather than gliclazide (Figure 9).

## 10. Conclusions

In this study, a novel silicone-grafted alginate platform was developed and characterized in terms of electrokinetic stability, surface active properties and rheological features. This new platform was employed in the drug delivery of gliclazide in the form of microcapsules produced by vibrational ionic gelation through the use of a Buchi B-390 encapsulator. Due to the biocompatibility of the PDMS and alginate employed in the formulation, these model microcapsules can be administered either orally or used to implant other drugs into a specific tissue/organ. To the best of our knowledge, this paper is the first to hybridize a crosslinked PDMS with alginate and further crosslink the entire platform with CaCl_2_ and TEOS to produce free-flowing gliclazide microcapsules that show high encapsulation efficiency for gliclazide, good mechanical stability and a tendency to sustain the release of their payload. Our future work will focus on optimising and improving the formulation for a better sustained release profile of the contained drug. 

## Figures and Tables

**Table 1:** The polymeric composition of each formula, Carr’s index and Hausner ratio.

Formulation	Na–Alginate (mg)	PDMS (mg)	Gliclazide (mg)	Final Formulation Volume (ml)	Cross Linker	Carr’s Index (Mean ± SD)	Hausner Ratio (Mean ± SD)
Control (N1)	4000	-	2000	To 150 mL H_2_O	CaCl_2_ 5%	12.12 ± 0.2	1.14 ± 0.003
N2	4000	4000	4000	To 150 mL H_2_O	CaCl_2_ 5%	10.72 ± 0.13	1.12 ± 0.002
N3	4000	2000	3000	To 150 mL H_2_O	CaCl_2_ 5%	11.42 ± 1.17	1.13 ± 0.015
N4	4000	4000	4000	100 mL 1% SDS + 50 mL H_2_O	(CaCl_2_ 5%+ TEOS 5%), (pH 1–3)	10.71 ± 0.13	1.12 ± 0.001
N5	4000	2000	3000	100 mL 1% SDS + 50 mL H_2_O	(CaCl_2_ 5%+ TEOS 5%), (pH 1–3)	10.79 ± 0.13	1.12 ± 0.002
N6	4000 in 100 mL H_2_O	4000 crosslinked with 4000 mg TEOS in acidified 50 mL 5% SDS	4000	150 mL	(CaCl_2_ 5%+ TEOS 5%), (pH 1–3)	9.23 ± 0.12	1.1 ± 0.001
N7	4000 in 100 mL H_2_O	2000 crosslinked with 2000 mg TEOS in acidified 50 mL 5% SDS	3000	150 mL	(CaCl_2_ 5%+ TEOS 5%), (pH 1–3)	10.68 ± 1.19	1.12 ± 0.014

**Table 2:** The characteristic FTIR peaks/bands for PDMS, TEOS, gliclazide and sodium alginate.

Ingredient	Characteristic FTIR Band/Peak	Represents	Reference
PDMS elastomer	Broad peak at 3295.5 cm^−1^	(-Si-OH), silanol group	[33,51,52,53]
	Sharp peak at 2962.2 cm^−1^	(-Si-CH_3_) group	
	Sharp peak at 1257.6 cm^−1^	Symmetric (-Si (CH_3_)_2_) stretching	
	Peak at 1022.9 cm^−1^	Asymmetric (-Si-O-Si-) stretching	
	Small peak at 888.4 cm^−1^	(-Si-OH), silanol group	
	Small peak at 856.2 cm^−1^	(-Si-CH_3_) group	
	Sharp peak at 791.8 cm^−1^	Asymmetric bending (-Si-(CH_3_)_2_)	
TEOS	Peaks at 2974.6 cm^−1^ and 2889.1 cm^−1^	-CH stretching in the ester group	[50,54]
	Small peaks at 1442.2 cm^−1^, 1390.6 cm^−1^ and 1295.9 cm^−1^	-CH asymmetric wagging/bending	
	Small peak at 1167.6 cm^−1^	CH3 rocking	
	Peak at 1099.6 cm^−1^	Si-O-C-O- asymmetric stretching of Si attached ethoxy group	
	Peak at 959.1 cm^−1^	-CH rocking	
Gliclazide	Peak at 3269.44 cm^−1^	-NH	[36,37,38,55,56,57]
	Peaks at 3190.9 cm^−1^ and 3110.39 cm^−1^	Aromatic CH	
	Peaks in the range (2947.86 cm^−1^–2836.22 cm^−1^)	CH stretching of the aliphatic perhydro-cyclopenta pyrrole ring	
	Sharp peak at 1707.26 cm^−1^	-C=O carbonyl stretch	
	Peak at 1595.88 cm^−1^	-NH bending	
	Peak at 1431.81 cm^−1^	Aromatic C=C stretching	
	Peak at 1345.36 cm^−1^	Asymmetric sulfonyl stretching (-S=O)	
	Peak at 1161.78 cm^−1^	Symmetric sulfonyl vibration(-S=O)	
	Peak at 1240.55 cm^−1^	Heterocyclic C-N ring stretch	
	Peak at 1086.79 cm^−1^	-C-O stretching	
	Peak at 995.3 cm^−1^	C=C bending	
	Peak at 917.95 cm^−1^	Aromatic *p* substitution, phenyl	
	Peak at 666.49 cm^−1^	Aromatic ring	
Na–alginate	Broad peak at 3241.7 cm^−1^	(-OH) stretching vibration	[28,58]
	Peak at 1594.75 cm^−1^	Asymmetric carboxylate stretching vibration	
	Peak at 1406.41 cm^−1^	Symmetric carboxylate stretching vibration	
	Peak at 1026.28 cm^−1^	-C-O-C- stretching vibration	

**Table 3:** DSC thermogram peaks identified for PDMS, SDS and crosslinked N6 and N7 dry films.

Ingredient	DSC Thermogram Peaks
PDMS elastomer	Endothermic peak at 205.3 °C
	Exothermic peak at 260.9 °C
SDS	Early endothermic dehydration peak at 112.1 °C
	Sharp peak at 198.5 °C (SDS melting point)
	Late peak at 275 °C
N6 Film	Endothermic peaks at 64.5 °C, 100.4 °C, 171.2 °C, 187.1 °C, 203.3 °C, 217.3, 239.7 °C and 264.9 °C
	Exothermic peak at 246.9 °C
N7 Film	Endothermic peaks at 71.3 °C,100.3 °C,164.9 °C, 183.4 °C, 200.7 °C, 218.7 °C, 234.6 °C and 262 °C
	Exothermic peak at 238.8 °C

**Table 4:** DSC thermograms of gliclazide, sodium alginate, control, N2, N4 and N6 microcapsules.

Ingredient	DSC Thermogram Peaks
Gliclazide	Sharp endothermic peak at 174.5 °C (melting point), reflects the pure crystalline state of the drug.
Na–alginate	Broad endothermic dehydration peak at 104.1 °C
	Exothermic decomposition peak at 253.1 °C
Control (N1) microcapsules	Endothermic peaks at 149.3 °C and 161.9 °C
N2 microcapsules	Endothermic peaks at 151.9 °C and 169.3 °C
N4 microcapsules	Endothermic peaks at 145 °C and 166.6 °C
N6 microcapsules	Endothermic peaks at 137.9 °C, 157.5 °C, 162.5 °C and 165.7 °C

## Data Availability

Authors can provide subject to request.
